# Drug incompatibilities in the adult intensive care unit of a university hospital

**DOI:** 10.5935/0103-507X.20160029

**Published:** 2016

**Authors:** Naiane Roveda Marsilio, Daiandy da Silva, Denise Bueno

**Affiliations:** 1 Integrated Multidisciplinary Care Residency Program, Hospital de Clínicas de Porto Alegre - Porto Alegre (RS), Brasil.; 2 Pharmaceutical Care Unit, Department of Pharmacy, Hospital de Clínicas de Porto Alegre - Porto Alegre (RS), Brazil.; 3 Postgraduate Program in Pharmaceutical Care, Faculdade de Farmácia, Universidade Federal do Rio Grande do Sul - Porto Alegre (RS), Brazil.

**Keywords:** Drug incompatibility, Administration, intravenous, Critical care, Pharmaceutical services, Intensive care units

## Abstract

**Objectives:**

This study sought to identify the physical and chemical incompatibilities among the drugs administered intravenously to patients admitted to an adult intensive care unit. We also aimed to establish pharmaceutical guidelines for administering incompatible drugs.

**Methods:**

This cross-sectional, prospective, and quantitative study was conducted from July to September 2015. Drug incompatibilities were identified based on an analysis of the patient prescriptions available in the hospital online management system. A pharmaceutical intervention was performed using the guidelines on the preparation and administration of incompatible drugs. Adherence to those guidelines was subsequently assessed among the nursing staff.

**Results:**

A total of 100 prescriptions were analyzed; 68 were incompatible with the intravenous drugs prescribed. A total of 271 drug incompatibilities were found, averaging 4.0 ± 3.3 incompatibilities per prescription. The most commonly found drug incompatibilities were between midazolam and hydrocortisone (8.9%), between cefepime and midazolam (5.2%), and between hydrocortisone and vancomycin (5.2%). The drugs most commonly involved in incompatibilities were midazolam, hydrocortisone, and vancomycin. The most common incompatibilities occurred when a drug was administered via continuous infusion and another was administered intermittently (50%). Of the 68 prescriptions that led to pharmaceutical guidelines, 45 (66.2%) were fully adhered to by the nursing staff.

**Conclusion:**

Patients under intensive care were subjected to a high rate of incompatibilities. Drug incompatibilities can be identified and eliminated by the pharmacist on the multidisciplinary team, thereby reducing undesirable effects among patients.

## Abstract (pt)

**Objetivos:**

Identificar as incompatibilidades físico-químicas entre medicamentos administrados por via intravenosa em pacientes internados em um centro de tratamento intensivo adulto, bem como realizar orientações farmacêuticas para a administração de medicamentos incompatíveis.

**Métodos:**

Estudo transversal, prospectivo, de caráter quantitativo, realizado no período de julho a setembro de 2015. As incompatibilidades foram identificadas a partir da análise das prescrições dos pacientes disponíveis no sistema *on-line* do hospital. Foi realizada uma intervenção farmacêutica por meio de orientações quanto à preparação e à administração dos medicamentos incompatíveis. Após, verificou-se a adesão dessas orientações por parte da equipe da enfermagem.

**Resultados:**

Foram analisadas 100 prescrições; destas, 68 apresentaram incompatibilidade entre os medicamentos intravenosos prescritos. Foram encontradas 271 incompatibilidades, com média de 4,0 ± 3,3 incompatibilidades por prescrição. As incompatibilidades mais frequentes foram entre midazolam e hidrocortisona (8,9%), cefepime e midazolam (5,2%) e hidrocortisona e vancomicina (5,2%). Os medicamentos mais envolvidos em incompatibilidades foram o midazolam, a hidrocortisona e a vancomicina. As incompatibilidades foram mais frequentes entre um medicamento administrado por infusão contínua com outro de forma intermitente (50%). Das 68 prescrições que geraram orientação farmacêutica, 45 (66,2%) foram totalmente realizadas pela equipe de enfermagem.

**Conclusão:**

Os pacientes em cuidados intensivos estiveram sujeitos a uma elevada ocorrência de incompatibilidades. As incompatibilidades medicamentosas podem ser identificadas e evitadas com a presença do farmacêutico na equipe multidisciplinar, diminuindo a ocorrência de efeitos indesejáveis ao paciente.

## INTRODUCTION

Intravenous therapy is commonly used in the hospital setting, and it is essential for patients who require rapid pharmacological effects or when barriers to oral drug administration exist. The choice of intravenous drug administration has inherent risks, including incompatibilities between administered drugs.^(1)^

Drug incompatibilities are physical and chemical reactions that occur *in vitro* between two or more drugs when the solutions are combined in the same syringe, tubing, or bottle.^(2)^ Physical reactions can cause visible changes, including precipitation; changes in color, consistency, or opalescence; or gas production. Chemical reactions are caused by molecular changes, and they are considered significant when more than 10% degradation of one or more of the solution's components occur. The major reason for differentiating these two types of incompatibilities is based on the contact time between one drug and the other. In the case of Y-site drug administration, the contact time is approximately 1 to 2 minutes depending on the infusion flow, whereas the contact time between drugs mixed in the same syringe or IV bag can last for hours or days, and chemical reactions can occur during that period.^(3)^ Drug incompatibilities can lead to reduced drug activity or inactivity, the formation of a new toxic or nontoxic active ingredient, increased toxicity of one or more of the involved drugs, and organoleptic changes.^(4)^

Numerous factors should be considered before concurrently administering two or more drugs to reduce the risk of incompatibility. The use of multilumen catheters might allow different intravenous drugs to be administered separately but simultaneously. Adjusting the drug administration schedules is also a key factor to be analyzed, as is whether the administration of a specific drug can be temporarily discontinued without compromising patient care while another medication is administered.^(5)^ Two incompatible drugs can also be administered consecutively, which makes it important to flush the infusion line with a compatible fluid between each administration.^(6)^ Another way to minimize the risk of incompatibilities includes the use of electronic prescriptions with alerts regarding the possible incompatibilities between the drugs prescribed. Some studies have already demonstrated that computerized alerts can influence drug prescriptions and avoid possible adverse events.^(7,8)^

Patients hospitalized in intensive care units (ICUs) are considered a high-risk group for the occurrence of incompatibilities because they commonly require the use of multiple drugs, most of which are administered intravenously. A common problem among these patients is the limited number of venous access routes, which complicates the safe administration of infusions that should ideally have a different access route for each drug. In these situations, most infusions occur using a Y-site connector, through which drugs are prepared separately but mixed in the lumen of the catheter before reaching the bloodstream. To enable simultaneous administration, the drugs should be physically compatible because chemical reactions require longer contact time for significant decreases in the drug concentrations to occur.^(9)^

The concomitant administration of incompatible drugs is a medication error and classified as a preventable adverse event that has the potential to cause patient harm.^(10)^ When evaluating prescription drug incompatibilities prior to their administration, the pharmacy staff can minimize these errors by guiding the nursing staff, thereby contributing to drug therapy efficacy and patient safety.

The objectives of this study were to identify the physical and chemical incompatibilities between the drugs administered intravenously to patients hospitalized at the Adult ICU of the *Hospital de Clínicas de Porto Alegre* (HCPA), establish pharmaceutical guidelines for administering incompatible drugs, and assess the adherence to those guidelines among the nursing staff.

## METHODS

This cross-sectional, prospective, and quantitative study was conducted in the ICU of the HCPA from July to September 2015.

Intravenous drug incompatibilities were identified based on an analysis of the patient prescriptions available in the hospital's online management system. The inclusion criteria were the prescriptions of patients with an ICU stay period equal to or longer than 24 hours but briefer than 72 hours and those containing four or more intravenous drugs. Only one prescription per patient was analyzed. Cases in which the drugs were prescribed for use only when necessary, patients under 18 years of age, and drugs that were unavailable in the database to assess their incompatibilities were excluded.

A previous drug incompatibility study conducted at the same hospital was used as the basis for sample calculation;^(11)^ and incompatibilities were identified in 78.5% of the prescriptions analyzed. The sample was estimated at 100 prescriptions, considering an 8% absolute margin of error and 95% confidence intervals.

Drug incompatibilities were assessed using the DrugDex^(r)^ Thomson Micromedex database accessed using the search engine of the online HCPA management system. After detecting incompatibilities in the prescriptions, pharmaceutical interventions were conducted in the form of written guidelines regarding drug preparation and administration, and these guidelines were attached to the bedside patient chart in a standardize form used by the Pharmaceutical Care Unit of the HCPA. The guidelines were established when combinations of incompatible, untested, or variable compatibility (depending on the concentration, solvent, or both) drugs were identified. These combinations often became incompatible when analyzed at the concentrations and solvents to be used by the patient.

Adherence to the guidelines among the nursing staff was assessed 24 hours after the pharmaceutical intervention. The statuses of full, incomplete (when at least one guideline was not followed), non-adherence, or non-applicability (when the patient died or was transferred to the ward before the guidelines could be evaluated) were recorded. The occurrence of any pharmacotherapy change precluding the guidelines from being properly followed was not considered as non-adherence.

The data collected were used to generate a database analyzed using Statistical Package for Social Science (SPSS) 22.0, and a descriptive analysis of the results was performed.

The Ethics Committee of the HCPA approved this study (Nº 10-0039). The data-use consent form was signed to ensure ethical aspects in compliance with Resolution 466/12 of the Brazilian National Health Council.

## RESULTS

Based on the inclusion and exclusion criteria adopted, 100 prescriptions for patients were analyzed from July to September 2015. A total of 63 (63%) patients were male. Patient age ranged from 20 to 91 years old, averaging 60.0 ± 15.5 years old. The length of hospitalization ranged from 1 to 42 days, averaging 9.8 ± 7.5 days. Table 1 shows the distribution of the reasons for patient hospitalization in the ICU, grouped by the system affected.

**Table 1:** Patient distribution by reason for hospitalization

**Reason for hospitalization**	**N**
Septicemia	35
Respiratory system disorders	26
Cardiovascular system disorders	13
Nervous system disorders	10
Renal system disorders	7
Hepatobiliary system disorders	5
Digestive system disorders	2
Hematologic system disorders	2
Total	100

A total of 1,019 prescription drugs were identified, averaging 10.2 ± 3.4 drugs per prescription. Of these drugs, 650 were intravenous, averaging 6.5 ± 2.4 drugs per prescription and ranging from 4 to 15 intravenous drugs per prescription.

At least one incompatibility was found in 68% of the 100 prescriptions analyzed. A total of 1,854 drug combinations were evaluated, and 271 (14.6%) incompatible, 372 (20.0%) untested and 1,211 (65.4%) compatible combinations were identified. Of the 271 incompatibilities identified, 108 showed different drug combinations. A mean of 4.0 ± 3.3 incompatibilities per prescription were observed (mean calculated based on the 68 prescriptions with drug incompatibilities).

The most common incompatibilities occurred between midazolam and hydrocortisone (8.9%), between cefepime and midazolam (5.2%), and between hydrocortisone and vancomycin (5.2%). Table 2 shows the incompatibilities most commonly found in the prescriptions analyzed. 

**Table 2:** Drug incompatibilities most commonly found among the prescriptions analyzed

**Drug incompatibilities**	**N (%)**
Hydrocortisone x midazolam	24 (8.9)
Cefepime x midazolam	14 (5.2)
Hydrocortisone x vancomycin	14 (5.2)
Cefepime x vancomycin	12 (4.4)
Omeprazol x vancomycin	11 (4.1)
Calcium chloride x hydrocortisone	10 (3.7)
Midazolam x omeprazol	10 (3.7)
Phenytoin x ranitidine	7 (2.6)
Phenytoin x midazolam	5 (1.9)
Phenytoin x noradrenaline	5 (1.9)
Hydrocortisone x vitamin B1	5 (1.9)
Sulfamethoxazole-trimethoprim x vancomycin	5 (1.9)
Phenytoin x fentanyl	4 (1.5)
Sulfamethoxazole-trimethoprim x fentanyl	4 (1.5)
Sulfamethoxazole-trimethoprim x hydrocortisone	4 (1.5)
Sulfamethoxazole-trimethoprim x ranitidine	4 (1.5)

**Figure 1:**
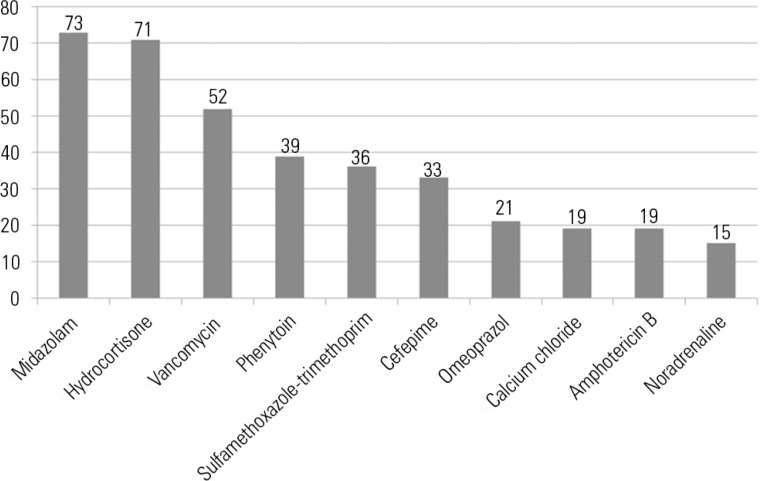
Frequency of drugs most commonly involved in the incompatibilities identified within the prescriptions analyzed.

Of the 58 different intravenous drugs analyzed, 45 were involved in incompatibilities, and the most common were midazolam, followed by hydrocortisone and vancomycin. Figure 1 shows the major drugs involved in incompatibilities in this study.

**Figure 2:**
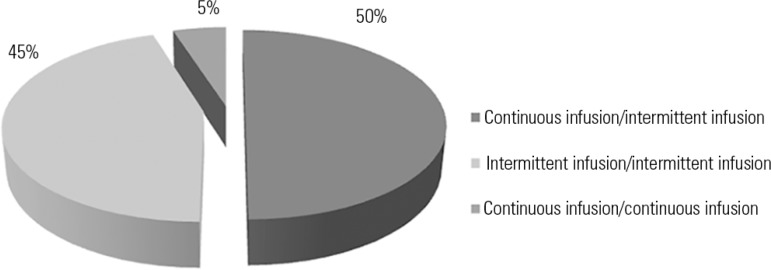
Type of intravenous drug administration involved in incompatibilities.

The analysis of the type of intravenous administration (continuous or intermittent infusion) showed that incompatibilities most commonly occurred between one drug administered via continuous infusion and another via intermittent infusion (50%). The other routes of administrations and the frequency rates of the drug incompatibilities are shown in figure 2. 

Of the total prescriptions analyzed, 68 pharmaceutical interventions were conducted by establishing guidelines for the preparation and administration of incompatible and untested drugs using a standardized form. Adherence to those guidelines is outlined in table 3.

**Table 3:** Adherence to guidelines by the nursing staff

**Answers**	**N (%)**
Full adherence to guidelines	45 (66.2)
Incomplete adherence to guidelines	15 (22.0)
Non-adherence to guidelines	0
Not applicable	8 (11.8)
Total	68 (100)

## DISCUSSION

In this study, incompatibilities were found in 68% of the prescriptions analyzed. This result is lower than the value observed in Moraes et al.^(11)^ who studied the adult ICU population of the HCPA and found incompatibilities in 78.5% of the prescriptions analyzed. Although a decreased prevalence of incompatibilities was found in the present study, this rate nevertheless remains high. The frequency of prescriptions with incompatibilities identified in this unit might be related to the numerous drugs prescribed to critically ill patients that are necessary given the complexity of their clinical conditions. The incidence of drug interactions increases exponentially with the number of drugs prescribed. A frequency ranging from 3% to 5% is estimated for patients who receive up to six drugs simultaneously, increasing to 20% among patients who receive ten drugs and reaches 45% among patients who receive 10 to 20 drugs.^(12,13)^ Thus, our study sample might be considered at high risk for the occurrence of drug interactions, especially drug incompatibilities, because a mean of 10.2 drugs were observed per prescription, most of which were intravenous drugs.

Our results regarding the number of incompatible combinations observed in this study (14.6%) are similar to those of Vogel Kahmann et al.^(14)^ who analyzed 78 different drugs and found that 15% of the combinations tested exhibited drug incompatibility reactions. Bertsche et al.^(15)^ and Gikic et al.^(16)^ found incompatibility rates of 7.2% and 3.4%, respectively, and the present study found a high prevalence of incompatibilities. The factors that might explain these differences in prevalence include the diversity of morbidity profiles among the samples that might change the drug therapy profile to be used and, consequently, the frequency of drug incompatibilities.

In this study, 20.0% of the combinations analyzed had no Y-site compatibility tests examined in the literature. A systematic review conducted at a hospital in Ottawa compiled 93 studies to evaluate the quality and quantity of the number of published studies on the physical and chemical stability of drugs commonly used in continuous infusion in the ICU. This review found that data were available regarding only 441 (54%) of the 820 combinations analyzed and concluded that Y-site compatibility studies for the drugs tested remain lacking, underlining the need to conduct further physical and chemical studies on this subject.^(3)^ The search strategy applied to obtain compatibility information among drugs has limitations. Databases, because of their periodic updating and inclusion of new stability and compatibility tests, are extensively used, although doubts have been raised about pairwise drug combinations that are untested or depend on infusion concentrations.^(17)^

Regarding the combinations of drugs most commonly involved in incompatibilities, the drug-use profile has changed over time. Moraes et al.^(11)^ found that the most common drug incompatibility occurred between piperacillin-tazobactam and midazolam. In this study, one of the most common drug incompatibilities occurred between midazolam and cefepime, and piperacillin-tazobactam was not recorded in any incompatibility identified. This between-study difference might be related to the fact that piperacillin-tazobactam was used less often at the study hospital, primarily because of cost-related drug-use restrictions, and was replaced by other antimicrobial drugs, including cefepime. This drug has a spectrum similar to piperacillin-tazobactam, but it is less expensive.

In this study, midazolam was the drug most commonly involved in incompatibilities, followed by hydrocortisone and then vancomycin. The high frequencies of these drugs in incompatibilities might be relative because they are widely used in the ICU and are therefore present in numerous prescriptions. The incompatibilities involving these drugs might be critical because they affect vital drugs such as sedatives, steroids, and antimicrobials.

Midazolam is widely used in the ICU as the first-choice drug for the continuous sedation of patients subjected to invasive procedures.^(18)^ This drug requires increased caution in its preparation and administration because it is commonly associated with serious adverse events.^(19)^

Corticosteroids have been used for more than 60 years as adjunctive treatments of infections to mitigate local and systemic inflammatory responses.^(20)^ These drugs are commonly used among critically ill patients, and a significant number of studies have demonstrated the benefits of using corticosteroids for patients in septic shock because they are associated with initial shock reversal, the mitigation of systemic inflammatory response indicators, and significant decreases in mortality.^(21,22)^

ICU patients receive injections and commonly require antimicrobial therapy. Approximately 20% to 40% of patients are estimated to receive antimicrobials to treat and prevent infections during hospitalization. The precipitation, inactivation, and change in stability caused by other drugs can reduce drug efficacy, leading to a low therapeutic index that is detrimental to antimicrobial therapy.^(23)^

Importantly, incompatibilities are strongly related to medication errors, which are key safety factors in patient care. Tissot et al.^(24)^ reported that drug incompatibilities account for 14.3% of all ICU medication errors, and Taxis and Barber^(25)^ demonstrated that drug incompatibilities are common in the ICU, possibly contributing to an up-to-25% increase in the rate of medication errors. Because medication errors are considered preventable adverse events, the multidisciplinary team accompanying the patient should participate in the drug therapy chain, from prescription to administration, to optimize pharmacotherapy and prevent such errors.^(26)^ As a team member, the clinical pharmacist should analyze the prescriptions and identify the problems that might affect the drug treatment, such as drug incompatibilities.

In this study, pharmaceutical interventions were conducted in all instances where prescriptions with drug incompatibilities were found via guidelines provided to the nursing staff regarding the preparation and administration of incompatible drugs. Several studies have already demonstrated a significant decrease in the number of adverse events caused by medication errors at institutions where pharmacists conduct medical staff interventions, especially in ICUs. Interventions decrease hospitalization costs and increase quality of patient care because they decrease the number of adverse events.^(27,28)^

A study conducted at an ICU in New York compared the number of drug interactions with and without the participation of the pharmacist in a review of the medical charts and prescriptions of hospitalized patients. That study demonstrated that having an on-call pharmacist led to a 65% decrease in the number of drug interactions, showing that improved identification and a lower number of significant drug interactions among ICU patients were possible because the pharmacist was involved, and the patients were evaluated daily.^(29)^

In the present study, pharmaceutical intervention contributed to the prevention and reduction of the occurrence of incompatibility reactions because adherence to guidelines (66.2%) led to the administration of incompatible drugs via different routes, at different times, or both. Incomplete adherence to guidelines (22.0%) was attributed to situations when one or more drugs were not administered via the indicated route or when any of the suggested times of drug administration was not accepted. No cases of non-adherence to the guidelines were observed. By performing a pharmaceutical intervention in the form of guidelines, the pharmacy department contributed to patient safety and promoted the increased integration of the pharmacist into the multidisciplinary team.

One limitation of this study is that it was conducted at an ICU, which has a specific morbidity profile more commonly associated with drug use that might prevent the generalization of our results to other populations. The analysis of incompatibilities involving the combination of only two drugs is another limitation of this study. However, the available data on the incompatibilities that might result by combining a greater number of drugs remain sparse, which would have prevented us from performing this study.

## CONCLUSIONS

Adults admitted to intensive care units are subjected to a high rate of drug incompatibilities that might be related to the numerous intravenous drugs prescribed. Importantly, a significant number of untested drug combinations still exists, highlighting the need for additional studies on this subject to provide increased safety regarding intravenous drug administration.

A pharmaceutical intervention enabled the prevention and reduction of drug incompatibilities, thereby increasing treatment efficacy and avoiding potential medication errors.
